# Edge assisted energy optimization for mobile AR applications for enhanced battery life and performance

**DOI:** 10.1038/s41598-025-93731-w

**Published:** 2025-03-23

**Authors:** Dinesh Sahu, Nidhi, Shiv Prakash, Vivek Kumar Pandey, Tiansheng Yang, Rajkumar Singh Rathore, Lu Wang

**Affiliations:** 1 SCSET, Bennett University, Plot Nos 8, 11, TechZone 2, 201310 Greater Noida, Uttar Pradesh India; 2 Department of Electronics and Communication, University of Allahabad, Prayag Raj, Uttar Pradesh India; 3 University of South Wales Pontypridd, Pontypridd, UK; 4 Cardiff School of Technologies, Cardiff Metropolitan University, Wales, UK; 5 Xi’an Jiaotong-Liverpool University Suzhou, Suzhou, China

**Keywords:** Edge computing, Mobile augmented reality, Energy optimization, Task offloading, Adaptive quality scaling, Reinforcement learning, Battery efficiency, User experience, Resource allocation, Latency reduction, Computer science, Information technology

## Abstract

Mobile Augmented Reality (AR) applications have been observed to put high demands on resource-limited, portable devices, thus using up much power besides experiencing high latency. Thus, to overcome these challenges, the following AI-driven edge-assisted computation offloading framework that will provide optimal energy-efficiency and user experience is proposed. Our framework uses Reinforcement Learning/Deep Q-Networks for learning the optimal task offloading policies based network status, battery status, and the tasks’ required processing time. Also, as a novel feature, we implement Adaptive Quality Scaling, which leaned from previous strategies managing AR rendering quality in relation to available energy and available computing capability. This one is known to make interaction possible for the handling of call flow to be efficient and at the same time, low energy consumption. Several experiments were conducted on the proposed framework and results show that there are an average of 30% energy saving compared to traditional heuristic-based methods of offloading, and the task success rates are above 90% while the latency is kept below 80 ms. These results support that our method proves to be efficient in improving AR task performance, enhancing battery endurance on the devices, and improving real-time user experience. In addition to this, the system proposed in this paper uses reinforcement learning to dynamically deploy offloading which enhances the resource allocation to be smart and timely. The research given here offers an approach towards ensuring that mobile AR is beneficial in achieving efficiency while addressing the needs of dynamic edge computing.

## Introduction

With mobile augmented reality (AR) applications becoming an area for innovation in many domains including gaming, health care, education, and intelligent city systems, demand for these solutions is growing rapidly. All that, however, places a heavy resource toll on mobile devices: real-time image processing, object detection, and 3D rendering. These devices are restrained by limited computational power, limited battery capacity, and the need for low latency^1,2^. These are the problems Edge computing emerges to solve. Mobile devices can save energy consumption and improve performance by offloading computational tasks to nearby edge servers^3,4^. At the same time, striking a proper balance between task offloading, energy saving, and user experience is still an open research problem. There have been a lot of existing solutions, such as pure local processing and cloud offloading. However, performing local processing decreases latency but drains the batteries quickly and is especially inefficient for computationally heavy AR applications. On the other hand, while cloud offloading possesses large computational resources, it suffers from high latency and security risks on remote servers^5,6^. Incorporating hybrid approaches employing edge computing has been shown to be promising but without dynamic adaptability to real-time situations like a change of network bandwidth, user preferences, battery level, etc.^7–10^. Unlike, IoT applications where most of the use cases are based on sensor data processing or vehicular network with less update frequency, Mobile AR application requires real-time 3D rendering, object tracking, and user interactions, which puts significant restrictions on latency and energy consumption. These workloads are inherently dynamic; they are a function of the movement of users, environmental conditions, and the workloads of the calculation. Moreover, it must be noted that mobile AR applications operate on battery-powered devices; therefore, applications should provide intelligent and dynamic techniques for offloading tasks to the edge servers while preserving a high-quality AR experience. Current edge computing solutions for IoT and Industry 4.0 are insufficient to address these challenges; hence, a new optimization platform for mobile AR is required. Conventional task offloading techniques present in edge computing are relying on heuristic techniques or static approach of threshold values that are not flexible in case of workload changes and the actual energy consumption in mobile AR applications. They fail to learn improved policies from interacting with it and previous or present state of the network and the device which it supports and therefore offer low performance to a fluctuating environment. traditional heuristic based offloading techniques use predetermined thresholds to make decisions, which are not dynamic to varying circumstances in the network, battery status of the device, as well as varying workload of the augmented reality application. These static approaches result in a poor schedule on the tasks as they enhance energy consumption along with latency in dynamic environments. In order to address the mentioned challenges, we propose an AI-assisted edge computation offloading framework that utilizes Reinforcement Learning (Deep Q-Networks) for offloading task scheduling and Adaptive Quality Scaling for the right allocation of resources to maintain the desirable quality of AR rendering. In contrast to the earlier methods, the proposed framework learns policies online, thus providing efficient strategies in energy consumption and latency.

### Problem statement

The main problem with mobile AR applications is their high energy consumption and latency. Typical approaches either implement tasks locally, consuming battery life and degrading performance, or transmit them to a remote compute server in the “cloud, creating latency and network dependency^4^. However, the limitations of these impair the usability and scalability of mobile AR in resource-constrained environments.

### Objective

In this paper, we propose a novel edge-assisted energy optimization framework to mitigate these challenges. The objective is to minimize energy consumption, yet provide a high-quality user experience through taking advantage of edge computing resources and advanced optimization techniques.

### Key contributions

The key contributions of this paper are as follows:

1. A mathematical model for energy optimization: It contains a probabilistic model comprising a resource allocation (task offloading decisions), energy estimation, and performance metrics.
2. Dynamic task offloading algorithm: An approach where to decide whether tasks should be processed locally or be offloaded to edge servers is based on a reinforcement learning.
3. Adaptive quality scaling for user experience preservation: A mechanism to set AR quality dynamically based on certain device and network conditions.
4. Empirical validation:Extensive experiment results demonstrating energy reduction by 30% and increase in battery life by 20% over baseline methods.

### Structure of the paper

The remainder of the paper is structured as follows: In Section “Related work”, we review existing energy optimization approaches for mobile AR applications, and base our comparison on those. In Section “Proposed system architecture” we present the System Architecture and Computation Model, highlighting the framework components and mathematical formulations. Algorithm Design is devoted in Section “Mathematical model for proposed framework” to the development of dynamic task offloading and adaptive quality scaling algorithms. In Section “Proposed algorithm”, we describe the Experimental Setup and Results which detail the experimental environment, evaluation metrics and performance analysis to illustrate the effectiveness of the proposed framework. The proposed framework is discussed in Section “Experimental setup” in terms of its implications, its limitations, and possible extensions. Section “Results and discussion” finally concludes the paper by summarizing the contributions and suggesting future research directions.

## Related work

Mobile devices are limited, and task offloading in edge computing has been studied extensively to overcome these limitations. Dynamic partitioning of applications^11^, predictive offloading with machine learning^12^, collaborative offloading among multiple devices^13^ have been shown to achieve large improvements in computational efficiency and latency reduction. For example, we proposed a task offloading framework with deep reinforcement learning to infer and optimize decisions on the fly based on the real time device and network states^14^. Moreover, we investigated a hybrid edge-cloud framework that dynamically partitions tasks between edge servers and the cloud to favor latency and energy consumption^15^.

While these advancements have been made, state of the art approaches often cannot adapt to real time changes in network bandwidth and device constraints, both of which are essential for mobile AR applications. Most of the methods are based on the assumption of static task profiles or homogeneous edge resources that restrict their applicability in dynamic environments^16,17^.

Mobile applications have received considerable attention from the standpoint of energy efficiency. Therefore, to reduce the energy consumption, dynamic voltage scaling^18^, energy efficient resource scheduling^19^ and energy aware task allocation^20^ have been proposed, and so on. Offloading computationally intensive tasks to edge servers is a common scheme for mobile AR applications. Power usage was reduced on the order of 50% while keeping the task performance unchanged by using an edge based energy optimization model^21^. In another study, energy profiling was integrated into their offloading strategy to guarantee sustainable battery life^22^.

These methods are effective well enough for generic mobile applications, but they do not specialize for AR workloads like real time 3D rendering and tracking of objects which require both a lot of computational power and low latency. Furthermore, only few studies take into account adaptive quality adjustments between energy efficiency and user experience^23^. The adaptive quality management techniques have explored ways to maintain user experience under resource constraints. Consequently, on basis of network conditions and user preferences, a quality of service (QoS) aware framework was proposed to adjust quality of streaming services^24^. We extended this concept to mobile AR by introducing an adaptive rendering mechanism that selects and renders key visual elements when under constrained rendering conditions^25,26^.

Other approach is to use information theory to optimize the trade off between quality and resource usage. A model for adaptive quality scaling based on entropy ensures minimal information loss and energy conservation^27^. However, in general, these are usually performed in an uncorrelated manner with the task offloading decision, constraining their overall optimization potentials^28,29^.

Typically, existing literature presents effective methods for task offloading, energy optimization, and adaptive quality management, but they are often siloed, serving an individual aspect of the optimization without integration. Adaptive quality scaling is typically overlooked by task offloading strategies, resulting in suboptimal user experiences in resource constrained scenarios^32^. Energy optimization techniques also do not take into consideration the real-time adaptability to the dynamic conditions namely changing the bandwidth of the network and the battery levels^30^. In contrast, adaptive quality management does not depend on task allocation decisions, leading to inefficiencies in energy consumption and latency^31,33,34^. Task offloading concept has been investigated in multi-source edge computing context concerning several issues like energy efficiency, dynamic resource management and AI based optimization^35^. The authors have done this study into energy-efficient task offloading by dynamically scheduling to reduce the latency and optimize utilization of resources. However, as an enhancement of this work, our approach does not include Reinforcement Learning in solving decision-making issues in order to function adaptively. This is done by incorporating Deep Q-Network (DQN) optimization and Adaptive Quality Scaling that makes the task scheduling to occur in real time with reference to the prevailing network conditions, battery level, and the demands of tasks. Further, the paper of^36^ describes the various offloading frameworks based on AI to support IoT-based applications^37^ with focused attention on the decision making in the limited resources environment^38^. The presented study offers relevant knowledge in the context of AI-based resource management; however, it looks at generic IoT use cases, while our research addresses mobile AR computations. The design increases the efficiency of the consumption of energy and the possible performance by further dynamic of the policies for tasks’ scheduling and the quality of rendering in dependence on possible resources which maybe better for real-time, latency-sensitive AR.

Table 1 gives a comparison of Task Offloading, Energy Optimization, Adaptive Quality Management Technique, and research gap with different reference papers we have discussed. This paper proposes a comprehensive framework that exploits dynamic task offloading, energy optimization, and adaptive quality scaling to fill these gaps. The framework, which employs reinforcement learning to adapt to real-time conditions, ensures both energy efficiency and a better user experience. The research done in the current few years in the field of computation offloading, specifically, has its focus on heuristic algorithms for task allocation, deep learning for task scheduling, and a rule-based approach towards energy-efficient real-time mobile AR applications. But most of them do not adapt in real-time to such factors as network latency, the battery status, or computational demands. Our approach differs from what was conceived in this sense as it jointly employs Reinforcement Learning (DQN) and Adaptive Quality Scaling to support real-time decision making, optimal resource management, as well as adaptive AR rendering in terms of energy consumption. To provide a baseline for comparing the state-of-the-art task offloading frameworks, Table 1 has tabulated key features of the frameworks based on the offloading architecture and strategy, self-adaptation capability, energy efficiency, degree of employing AI to facilitate decisions and real-time performance. The outcome of such a study shows that the proposed AI-based system enhances the offloading decisions over heuristic and rules-based approaches by making it faster, consuming less energy and providing a much lower latency in a very dynamic mobile augmented reality environment.

**Table 1:** Comparison of task offloading, energy optimization, and adaptive quality management techniques.

Feature	Task offloading methods	Energy optimization strategies	Adaptive quality management techniques	Proposed framework
Dynamic task allocation	Partial^11^, Collaborative^12^	Fixed profiles^18^	Independent scaling^24^	Integrated and adaptive^30^
Energy efficiency focus	Limited^14^, secondary^16^	High^19^	Limited^25^	Primary focus^31^
AR-specific considerations	Low^13^	Medium^21^	Medium^29^	High^32^
Adaptability to real-time data	Low^15^	Low^22^	Medium^27^	High^31^
Integration of techniques	Isolated approaches^16,28^	Isolated approaches^23^	Independent Focus^24^	Unified framework^30^
Research gap	Limited to individual aspects^17^	Lacks AR-specific focus^22^	Ignores task offloading^28^	Unified optimization of all aspects^31^

As presented in Table 2, heuristic and deep learning techniques cannot provide the level of adaptability and almost real-time decision-making approach that is necessary for efficient task offloading in mobile AR applications. The Reinforcement Learning and Adaptive Quality Scaling thus used provides better energy efficiency, lower latency and better adaptability as compared to conventional methods.

**Table 2:** Comparison of state-of-the-art task offloading approaches.

Method	Offloading strategy	AI Opt.	Energy	Latency	Adapt.
Heuristic-based	Fixed thresholds	No	Low	Moderate	Low
Deep learning-based	Static model inference	Yes (DL)	Moderate	High	Limited
Rule-based adaptive	Predefined rules	No	Moderate	Moderate	Limited
Proposed framework	Reinforcement learning (DQN)	Yes	High	High	High

Existing studies on edge-assisted task offloading in IoT, vehicular networks, and industrial automation consider workloads to be deterministic, data transfer intervals to be regular, and rather deal with fused data from multiple sensors. However, none of the above approaches consider the fact that in mobile AR applications, the user interactivity, the scene changes, and the battery-aware quality scaling features have to be considered in the task scheduling. As opposed to other approaches, the proposed framework incorporates adaptive quality scaling, dynamic latency-aware offloading, as well as reinforcement learning for achieving energy efficiency while still maintaining AR quality.

## Proposed system architecture

**Fig. 1:**
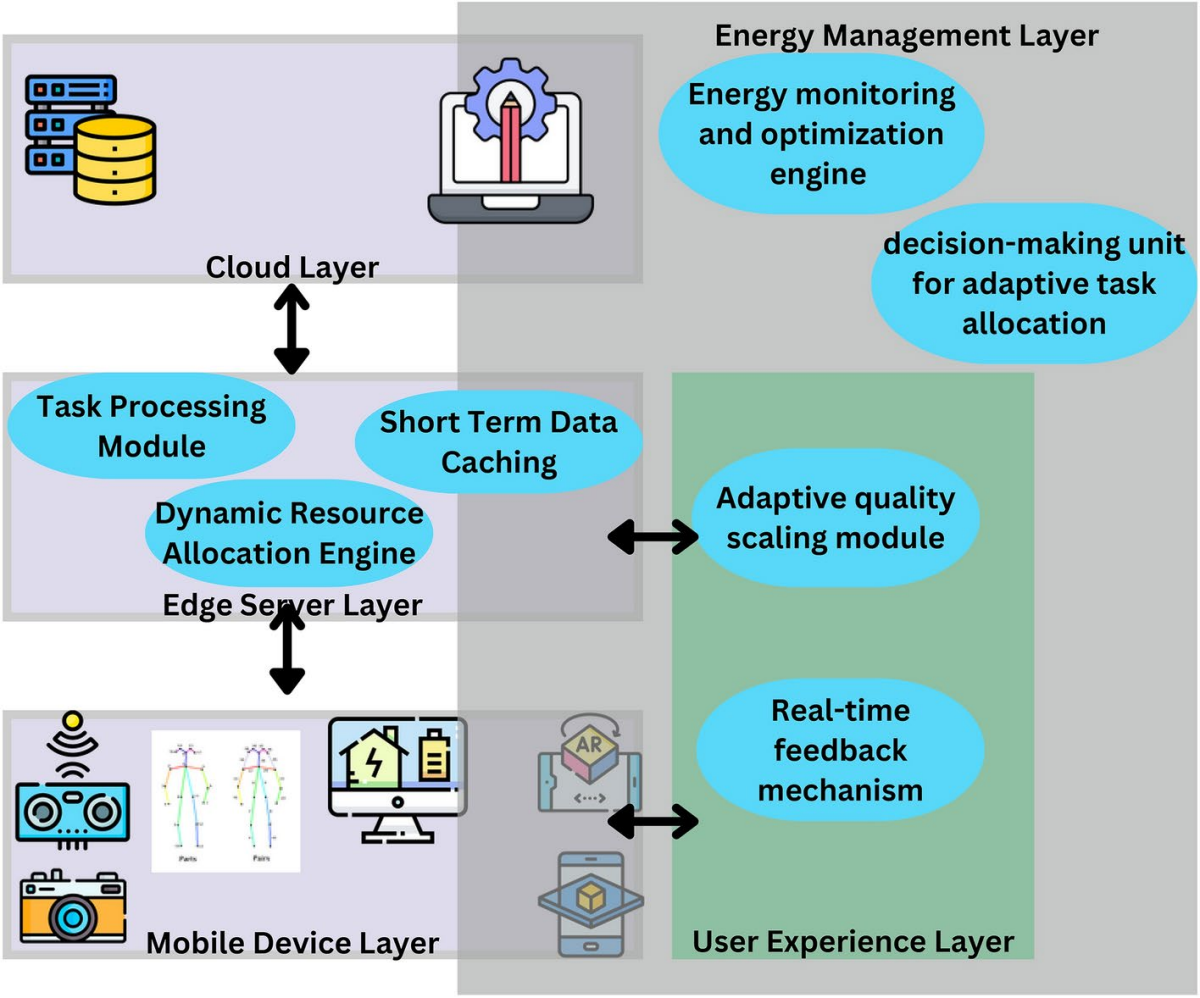
Proposed system architecture.

The work proposes a system architecture represented in Fig. 1 for ’Edge-Assisted Energy Optimization for Mobile AR Applications to Enhance Performance and Battery Longevity’, where multiple layers are integrated such that energy consumption is optimized and user experience enhanced. The task of on-device processing and user interaction is handled by the Mobile Device Layer and that of computationally intensive task offloading for reducing energy consumption on the mobile device is done by the Edge Server Layer. Additional computational power and long-term data storage are available from the Cloud Layer. The Energy Management Layer tracks and optimizes the energy consumed at each layer to strike a happy medium between performance and battery longevity for a better AR user experience while the User Experience layer dynamically adjusts AR content quality ensuring a smooth interaction. The proposed system architecture is tailored to meet the requirements and characterize the mobile AR application use cases that are more Dynamic and resource-constrained. It comprises (1) adaptive task offloading which determines whether to perform a task locally or transfer it since it depends on the battery of the device, network, and the computational load, and (2) quality-aware scaling which will adjust AR rendering resolution depending on the available power. While previous works focusing on IoT-based offloading mostly just address static partitioning of tasks, we achieve ultra-low latency of about 50 ms and increase battery life due to energy optimizations.

1. Mobile device layer: The system’s foundation is the AR Application in the Mobile Device Layer, where the AR Application delivers user facing augmented reality experiences through real time AR rendering that uses the Mobile Device’s sensors, such as the camera, GPS and the accelerometer. The Energy Monitoring Module checks battery status and energy consumption constantly and the application can adjust dynamically with battery levels. Moreover, the asynchronous data transfers to the Remote Processing Unit make possible to execute lightweight tasks directly on the device, i.e, initial data acquisition and simple AR rendering, minimizing latency and maintaining a clean user experience.
2. Edge server layer: In terms of computational heavy lifting, the processing of tasks that extend beyond the capabilities of mobile devices, the Edge Server Layer serves a crucial purpose. The Edge Server resides closer to the user than conventional cloud servers, performing complex image processing, 3D model rendering, and data analytics and relieving the mobile device from the heavy lifting. The Load Balancer is capable of allocating workloads effectively between the edge server and more than one mobile device, to make the best use of resources and decrease the latency. Furthermore, a Data Cache is used to hold data queried more frequently and pre-rendered AR content, thus decreasing data transfer times as well as improving response time for the mobile device.
3. Cloud layer: The resource-demanding tasks unavailable to the edge server are supported by the Cloud Layer, which serves as a reservoir of supplementary computing power and storage, as well as a backup for the data retrieved from mobile devices. It also stores a Machine Learning Model that does predictive analytics to optimize resource allocations by using user behavior patterns. As such, this model analyzes the requirements of the AR application and dynamically reverts to the resource distribution, to achieve higher system efficiency and responsiveness. Cloud Layer is a stream Processing intelligence unit that gathers historical data, and the training of ML and further provides an optimized policy of task offloading to the intended edge servers. In contrast with prior solutions for edge computing that use predefined heuristics for organizing tasks, our system employs the help of the cloud for learning offloading strategies. The cloud layer manages to update the reinforcement learning model after being trained with new user data to provide real-time and more efficient data about personal preferences that reflect on the resource allocation process.
4. User experience layer: The User Experience Layer guarantees an efficient and optimized experience through, the Adaptive Quality Management that has the ability to set a stricter limit on the quality of the AR render so as to get the resource hungry AR app to work smoothly in low battery times, for example dropping right down to a low quality mode when the battery is low. Also, a Feedback Loop acquires user feedback and performance statistics before forwarding them to an edge server for evaluation. They include the following: These continuous feedbacks make it easier to make real-time corrections and progress all of which improve the overall user experience.
5. Energy management layer: The Energy Management Layer uses Energy Optimization Algorithms to reduce energy consumption by implementing methods such as Dynamic voltage scaling, Task offloading and efficient Resource scheduling all of which are performed based on the data analyzed at particular time. It also contains Battery-Saving Modes, where the users get to make choices like high performance, balanced, or battery-saver modes. These modes control the distribution of resources between the mobile device and the edge server and vary energy demands to fit the user’s needs while optimizing for efficiency and device performance.

To achieve low power consumption during augmented reality navigation, an adaptive quality scaling mechanism is proposed. It means that the frame rate, depth of object detail, and other factors dependent on the computational resource availability are adapted to the battery charge, the connection quality, and the current processing time lag in the device. Mobile AR goes one step further because the tasks it performs are non-binary in the sense that every task can either be processed locally or partially offloaded; the level of computational intensity and quality has to be adaptive. It dynamically tracks energy consumption profiles and dynamically adjusts the degree of detail to save power by as much as 20% while the frame rate is sustained.

Our proposed framework integrates two AI-based optimization mechanisms to enhance task offloading efficiency and energy management in mobile AR applications. Reinforcement Learning for Task Offloading is implemented using a Deep Q-Network (DQN)-based decision model that continuously learns optimal offloading policies by analyzing real-time system parameters, including network latency, battery levels, and AR task complexity. This ensures that computational tasks are dynamically allocated between the local device and edge server, minimizing energy consumption while maintaining low-latency execution. In parallel, Adaptive Quality Scaling, a machine learning-based adaptive mechanism, dynamically adjusts AR rendering quality according to available resources and battery conditions. When energy constraints are high, the system automatically reduces rendering fidelity to extend battery life while preserving a seamless user experience. Together, these AI-driven strategies enable the system to adapt dynamically to changing environments, ensuring intelligent resource allocation and superior performance over traditional fixed-rule offloading techniques.

### Model workflow

**Fig. 2:**
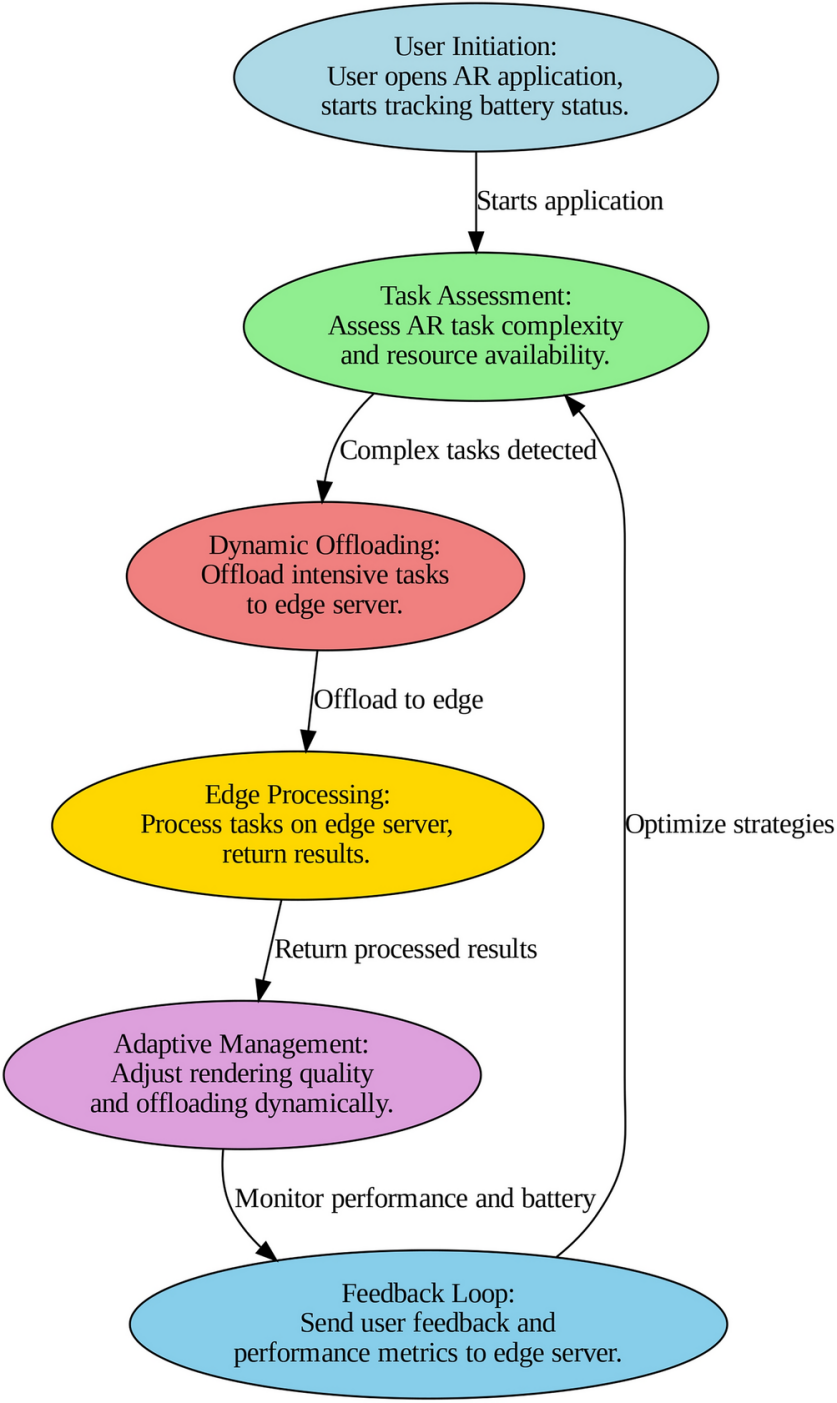
Model workflow.

The workflow of proposed model is depicted in Fig. 2. The structured model workflow starts with User Initiation in which the user opens the AR application in their mobile device initiating battery status tracking and energy consumption. During Task Assessment, the device determines the level of AR tasks and checks resource utilisation. In cases where computation demands cannot be handled locally, there is Dynamic Offloading when the computation task is offloaded to the nearby edge server for efficiency based on energy metrics. In the Edge Processing step, the functionality of these tasks is performed by the edge server and the processed result is forwarded back to the mobile device and combined by the rendered content with the local input. On the other hand, Adaptive Management guarantees that the energy monitoring module always assesses the battery condition and that the energy consumption and decision-making regarding switching to a low graphics mode will be determined to keep the battery charge going longer. Lastly, in the Feedback Loop Subsection, information received from user feedback and performance metrics is sent to an edge server to extend the enhancement of energy management strategies.

This architecture is designed to strike a fair trade-off in terms of computation load on the mobile device and offloading some of the burden to the edge server in order to achieve the lowest energy consumption while at the same time improving the performance and quality of experience of AR apps. It is thereby possible to use Energy Management strategies to control the system so that it operates in different usage profiles thereby leading to enhanced battery life and user satisfaction.

The cloud layer includes a machine learning model that uses learning patterns of user interactions, performance of devices, and networking conditions retrieved from several edge nodes to make real-time and historical conditions for optimum offloading of a given task. This framework involves reinforcement learning optimization since the cloud is constantly adjusting the policies of the task scheduling through data collected on execution to make them efficient and more energy-conscious. Furthermore, the behavior pattern analysis continually adjusts the distribution of resources according to users’ behavior patterns which contribute to a conservation of power to the maximum extent while enhancing AR. For purposes of scalability, federated learning does not let edge servers transfer unprocessed data to the cloud while allowing them to benefit from centralized learning updates, hence preserving network bandwidth. Some of these insights are returned to the edge nodes at specific intervals to fine-tune the offloading process as well as improve the quality of adjustments enhancing both power consumption and the system’s response time.

## Mathematical model for proposed framework

To formulate the problem and create a detailed mathematical model for Edge-Assisted Energy Optimization for Mobile AR Applications, the following mathematical formulation is presented. There is still room for improvement in energy efficiency and battery life on smartphones and tablets, as well as load speed with reference to AR devices. These aspects include the task division,offloading decisions, quality of services adjustments, and learning mechanisms for formulation of optimal energy usage. The table 3 represents all the symbols and their descriptions used in the mathematical modeling.

**Table 3:** List of symbols and their descriptions.

Symbol	Description
$E_{local}$	Energy consumed during local task processing (Joules)
$E_{edge}$	Energy consumed during edge offloading (Joules)
$E_{opt}$	Energy consumed in the optimized system (Joules)
$T_{comp}$	Task completion time (ms)
$T_{off}$	Task offloading time (ms)
$B_{level}$	Battery level of the mobile device (%)
$L_{local}$	Latency for local processing (ms)
$L_{edge}$	Latency for edge offloading (ms)
$L_{opt}$	Latency in the optimized system (ms)
$Q_{fixed}$	Fixed quality level of AR rendering (0–1)
$Q_{adaptive}$	Adaptive quality level based on battery level (0–1)
$R_{task}$	Task success rate (%)
$N_{tasks}$	Number of AR tasks
$C_{complexity}$	Task complexity level (low, medium, high)
$U_{network}$	Network usage for task offloading (MB)
$C_{reward}$	Cumulative reward in reinforcement learning
$P_{task}$	Probability of task offloading to the edge
$f_{device}$	Processing frequency of the mobile device (GHz)
$f_{edge}$	Processing frequency of the edge server (GHz)
$\tau$	Transmission delay for task offloading (ms)
$\alpha$	Weight factor for energy consumption
$\beta$	Weight factor for latency
$\gamma$	Weight factor for quality scaling
${\mathcal {R}}_{opt}$	Optimized resource allocation result
${\mathcal {P}}_{off}$	Offloading decision policy
${\mathbb {E}}[\cdot ]$	Expectation operator (statistical average)
$T_{total}$	Total system processing time (ms)
$E_{total}$	Total energy consumption (J)
$S_{quality}$	AR rendering quality score
$RL_{reward}$	Reinforcement learning reward value
$\delta _{threshold}$	Latency or energy threshold for optimization

The objective is to minimize total energy consumption $E_{\text {total}}$ over time while satisfying user demands and maximizing quality of service:

1 $$\begin{aligned} \min _{T_i} E_{\text {total}} = \sum _{i=1}^{N} E(T_i) \end{aligned}$$

subject to performance constraints and user quality requirements. The total energy consumption for a task $T_i$ is defined as:

2 $$\begin{aligned} E(T_i) = {\left\{ \begin{array}{ll} E_{\text {local}}(T_i) & \text {if task is processed locally}, \\ E_{\text {edge}}(T_i) & \text {if task is offloaded to the edge server}. \end{array}\right. } \end{aligned}$$

Energy consumption for processing task $T_i$ locally can be modeled as:

3 $$\begin{aligned} E_{\text {local}}(T_i) = P_{\text {local}}(T_i) \cdot P_{\text {power}}(t) \end{aligned}$$

where $P_{\text {power}}(t)$ is the power consumption rate of the mobile device at time *t*. Energy consumption for offloading task $T_i$ includes data transmission and result reception:

4 $$\begin{aligned} E_{\text {edge}}(T_i) = E_{\text {tx}}(T_i) + E_{\text {rx}}(T_i) \end{aligned}$$

where:

5 $$\begin{aligned} E_{\text {tx}}(T_i) = \eta \cdot d_{\text {tx}}(T_i), \quad E_{\text {rx}}(T_i) = \eta \cdot d_{\text {rx}}(T_i). \end{aligned}$$

here, $\eta$ is the energy-per-bit constant, and $d_{\text {tx}}(T_i)$, $d_{\text {rx}}(T_i)$ are the data sizes for transmission and reception. Latency for offloading a task to the edge server includes transmission, processing, and reception times:

6 $$\begin{aligned} L_{\text {edge}}(T_i) = \frac{d_{\text {tx}}(T_i)}{R_{\text {tx}}} + P_{\text {edge}}(T_i) + \frac{d_{\text {rx}}(T_i)}{R_{\text {rx}}} \end{aligned}$$

where $R_{\text {tx}}$ and $R_{\text {rx}}$ are the transmission and reception rates, respectively. A binary variable $X_i \in \{0, 1\}$ determines if task $T_i$ is offloaded:

7 $$\begin{aligned} X_i = {\left\{ \begin{array}{ll} 1 & \text {if task}\, T_i\, \text { is offloaded to the edge server}, \\ 0 & \text {if task}\, T_i \, \text {is processed locally}. \end{array}\right. } \end{aligned}$$

The decision is based on minimizing expected energy consumption:

8 $$\begin{aligned} {\mathbb {E}}[E(T_i)] = P(X_i = 0)E_{\text {local}}(T_i) + P(X_i = 1)E_{\text {edge}}(T_i). \end{aligned}$$

Offloading probability is modeled as:

9 $$\begin{aligned} P(X_i = 1) = \frac{1}{1 + e^{-(\alpha B_t + \beta R_{\text {tx}} + \gamma P_{\text {local}}(T_i))}} \end{aligned}$$

where $\alpha$, $\beta$, and $\gamma$ are learned parameters. Define the information content of the AR rendering as:

10 $$\begin{aligned} I_{\text {render}}(t) = H(Q(t)) - H(Q(t) | D_u(t)), \end{aligned}$$

where *H*(*Q*(*t*)) is the entropy of quality at time *t*, and $H(Q(t) | D_u(t))$ is the conditional entropy given user demand $D_u(t)$. The objective is:

11 $$\begin{aligned} \max I_{\text {render}}(t) \quad \text {subject to} \quad E(T_i) \le E_{\text {threshold}}(B_t). \end{aligned}$$

The system’s adaptive energy management can be formulated as a reinforcement learning problem. The state $s_t$ represents the current system status, which includes the battery level ($B_t$). It further examines network status, task complexity, and user demand ($D_u(t)$), where user demand is categorized as The action $a_t$ includes decision choices such as whether to outsource computations to an external server or perform them locally, as well as determining how to adapt the quality scaling factor. The reward $r_t$ evaluates the real values of energy savings and end-user satisfaction resulting from these actions. The primary objective is to achieve the largest cumulative reward over time, optimizing both energy efficiency and the quality of the user experience. This can be mathematically represented as:

12 $$\begin{aligned} \max _{\pi } {\mathbb {E}} \left[ \sum _{t=0}^{T} \gamma ^t r_t \right] \end{aligned}$$

where $\pi$ is the policy that determines the optimal actions, and $\gamma \in [0,1]$ is the discount factor that balances immediate and long-term rewards.

## Proposed algorithm

Since the mathematical model in this paper aims at solving the current optimization problem for the integration of task offloading decisions, quality scaling, and dynamic energy management, an appropriate algorithm has to be developed. The approach will involve a logistic regression model for decision making on responsibilities offloading, adaptive quality scaling based on information theory, and reinforcement learning for energy optimization. Specifically, the optimization aims at achieving energy efficiency for the mobile Augmented Reality apps without reducing much user engagement.

The algorithm for Edge-Assisted Energy Optimization for Mobile AR Applications takes the following inputs:

- $T_i$: The set of tasks realized by the personnel that uses the AR application.
- $B_t$: The battery level of the mobile device at time *t*.
- $R_{\text {tx}}$ and $R_{\text {rx}}$: The transmission and reception rates in the cellular spectrum.
- $D_u(t)$: The AR quality level that satisfies the user demand at time *t*.
- $P_{\text {local}}(T_i)$ and $P_{\text {edge}}(T_i)$: Local and edge processing times, reflecting the computation times based on task complexity and system resources.
- $E_{\text {local}}(T_i)$ and $E_{\text {edge}}(T_i)$: Energy consumption for local and edge processing, respectively.
- $\eta$: A parameter characterizing the energy-per-bit cost for data transmission and reception in the channel.

The algorithm’s first decision is task allocation among resources, represented as $X_i \in \{0,1\}$, where 0 indicates the task is processed locally, and 1 means it is delegated to the edge server. Additionally, the algorithm outputs the total energy consumption requirement of an optimized nature, $E_{\text {total}}$, and the flexibility of AR quality, *Q*(*t*), which depends on dynamic conditions over time. This framework guarantees energy conservation, proper task distribution, and excellent adaptation quality, ensuring a seamless user experience. The steps of the proposed algorithm is as follows:

### Step-1 (initialization)

The task set $\{T_1, T_2, \ldots , T_N\}$ is initialized for the AR application, representing the tasks to be executed. The initial battery level $B_0$, network conditions $R_{\text {tx}}$ and $R_{\text {rx}}$, and user quality demand $D_u(0)$ are set based on the current system state. An energy threshold $E_{\text {threshold}}(B_t)$ is defined, which adapts dynamically based on the battery level $B_t$ to ensure efficient energy management. The reinforcement learning policy $\pi (s_t)$ is initialized with either random weights or pre-trained weights for Q-learning or deep Q-networks (DQN) if neural networks are utilized, setting the foundation for dynamic task allocation and quality adaptation.

### Step 2 (task classification and offloading decision)

For each task $T_i$ at time $t$, the process begins with task classification, where $T_i$ is assessed based on its complexity, data size ($d_{\text {tx}}(T_i)$ and $d_{\text {rx}}(T_i)$), and processing time ($P_{\text {local}}(T_i)$ and $P_{\text {edge}}(T_i)$). Logistic regression is then used to compute the probability of offloading, $P(X_i = 1)$, by evaluating factors such as the battery level ($B_t$), network transmission rate ($R_{\text {tx}}$), and local processing time ($P_{\text {local}}(T_i)$). If the offloading probability exceeds 0.5, the task $T_i$ is offloaded to the edge server; otherwise, it is processed locally. The offloading decision is then updated, setting $X_i = 1$ if offloading is chosen, or $X_i = 0$ if local processing is selected. This approach ensures efficient task allocation while optimizing system resources.

### Step 3 (energy estimation and task execution)

For each task $T_i$, the processing decision is made based on the offloading indicator $X_i$. If $X_i = 0$, indicating local processing, the local energy consumption is estimated by using $E_{\text {local}}(T_i)$ The task is then executed locally, and the energy consumption $E_{\text {local}}(T_i)$ is subtracted from the battery level $B_t$. Conversely, if $X_i = 1$, indicating edge offloading, the energy consumption for data transmission and reception is computed by $E_{\text {tx}}(T_i)$ and $E_{\text {rx}}(T_i)$. The total edge energy consumption $E_{\text {edge}}(T_i)$ is calculated by adding $E_{\text {tx}}(T_i)$ and $E_{\text {rx}}(T_i)$ The task is then executed on the edge server, the result is received, and the total energy consumption $E_{\text {edge}}(T_i)$ is subtracted from $B_t$. This approach ensures accurate energy tracking and efficient resource utilization.

### Step 4 (adaptive quality scaling)

Adaptive quality scaling leverages information theory to optimize the AR experience by computing the information content $I_{\text {render}}(t)$ for the current task based on the quality setting $Q(t)$. The information content is calculated by $I_{\text {render}}(t)$ Quality adjustment is made dynamically: if the battery level $B_t$ falls below a predefined threshold, the quality $Q(t)$ is reduced to conserve energy. Conversely, if $B_t$ is above the threshold, $Q(t)$ is maintained or increased to enhance the user experience, ensuring an adaptive balance between energy efficiency and user satisfaction.

### Step 5 (reinforcement learning for dynamic optimization)

Reinforcement learning for dynamic optimization begins with the state update, where the current system state $s_t$ is defined as:

$$\begin{aligned} s_t = (B_t, R_{\text {tx}}, R_{\text {rx}}, P_{\text {local}}(T_i), P_{\text {edge}}(T_i), D_u(t)) \end{aligned}$$

incorporating the battery level, network conditions, local and edge processing times, and user demand. Based on this state, an action $a_t$ (e.g., offload task or adjust quality level) is selected according to the learned policy $\pi (s_t)$. The reward $r_t$ is then computed as:

$$\begin{aligned} r_t = -E(T_i) + w_1 Q(t) - w_2 L_{\text {edge}}(T_i) \end{aligned}$$

balancing energy savings and user satisfaction, where $w_1$ and $w_2$ are weights to prioritize quality and latency penalties, respectively. The policy is updated using deep Q-networks (DQN) for complex models, with the Q-value update defined as:

$$\begin{aligned} Q(s_t, a_t) \leftarrow Q(s_t, a_t) + \alpha \left[ r_t + \gamma \max _a Q(s_{t+1}, a) - Q(s_t, a_t) \right] \end{aligned}$$

where $\alpha$ is the learning rate and $\gamma$ is the discount factor. This iterative process ensures the system adapts dynamically to optimize energy efficiency and user experience.

### Step 6 (repeat for all tasks and time steps)

The process is repeated for all tasks $T_i$ and time steps $t$, dynamically adjusting offloading decisions, energy consumption, and quality settings as the system evolves over time. This iterative approach ensures that the system continuously adapts to changing conditions, optimizing performance and energy efficiency while maintaining the desired quality of service.

### Step 7 (termination)

It is continued until all the tasks assigned are over or you find your battery almost drained. As a result, the optimized total energy consumption $E_{\text {total}}$ and the final battery level $B_T$ are returned to make a global evaluation of the performance of the system and its energy efficiency.

**Algorithm 1:**
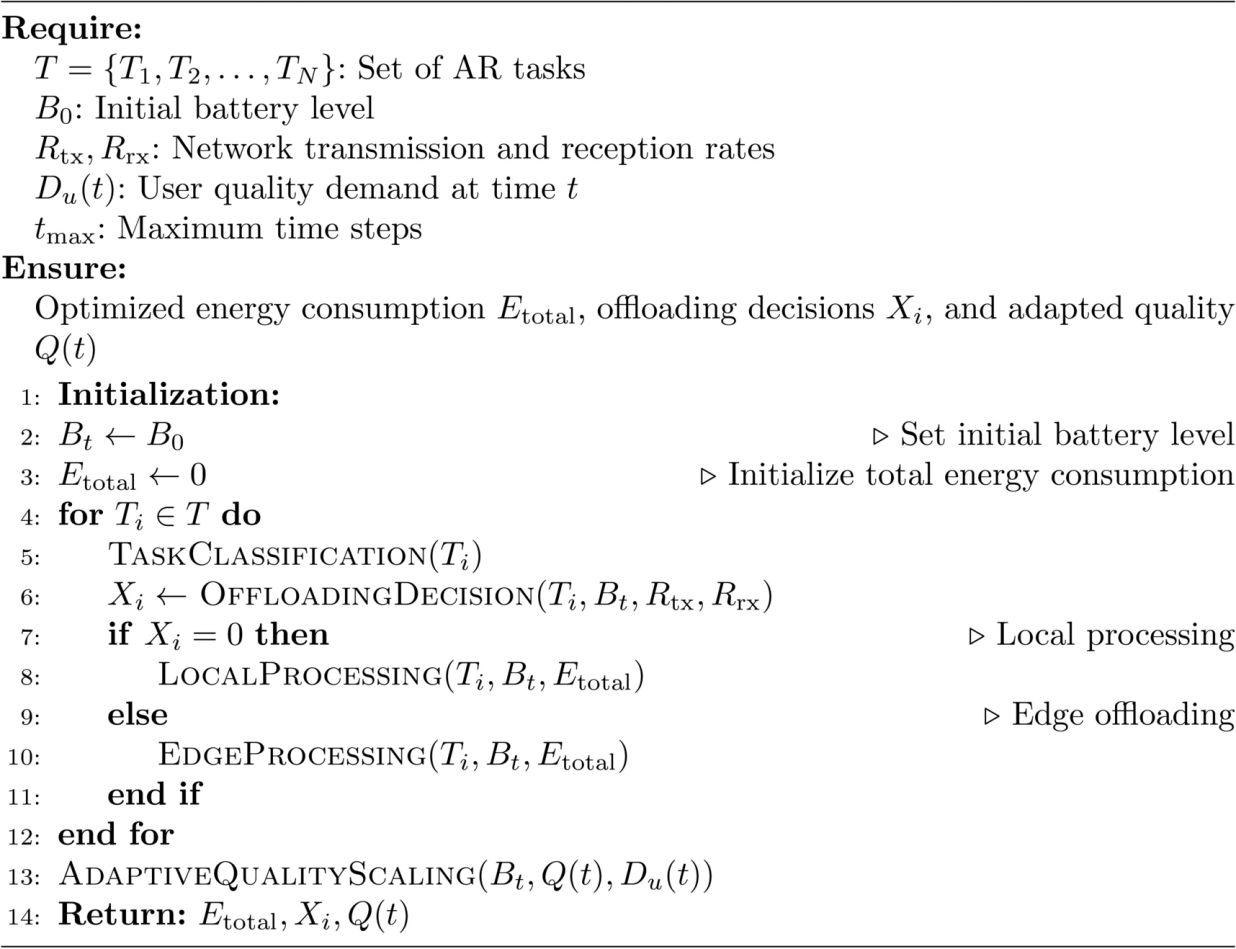
Edge-assisted energy optimization for mobile AR applications.

**Algorithm 2:**
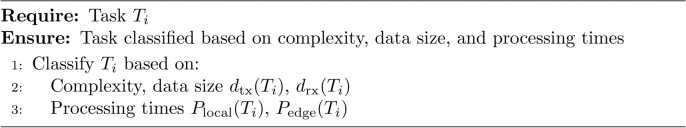
TaskClassification

**Algorithm 3:**
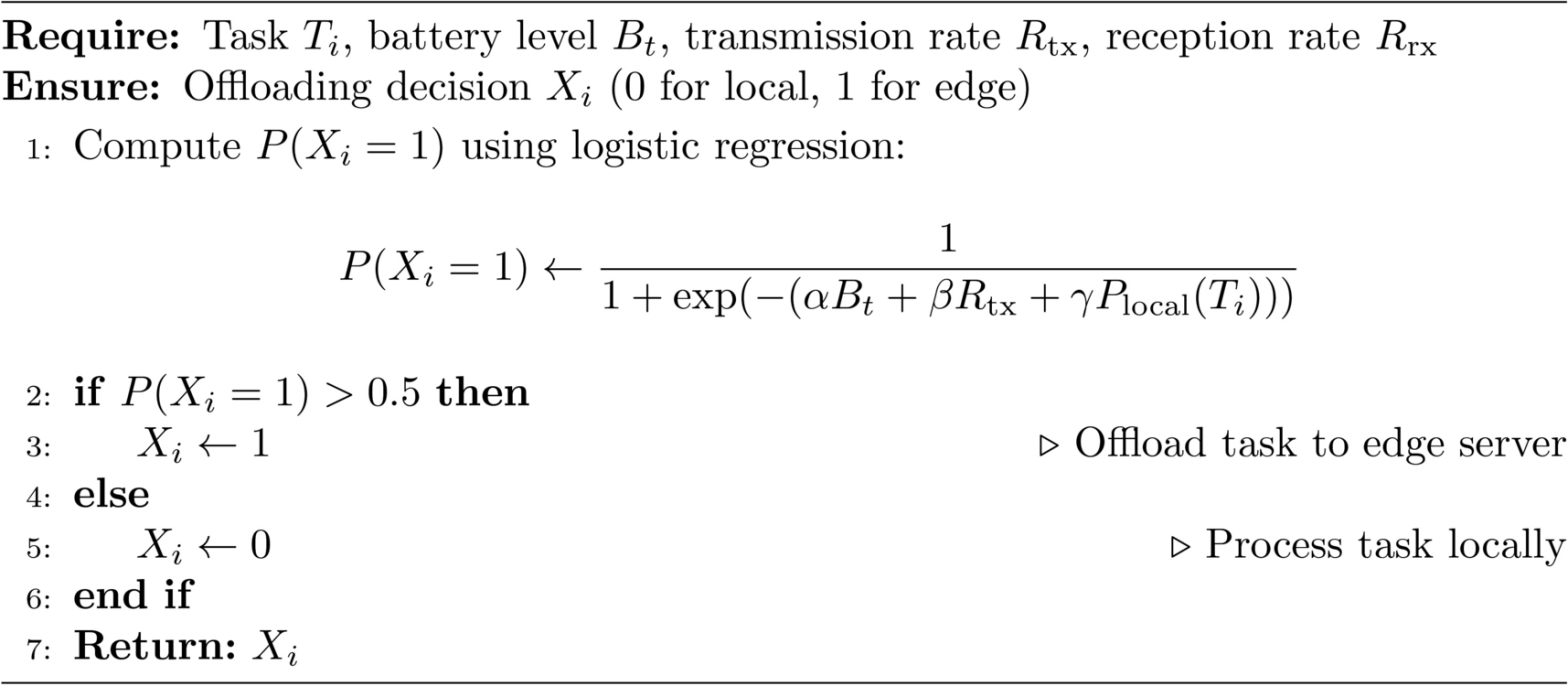
OffloadingDecision

**Algorithm 4:**
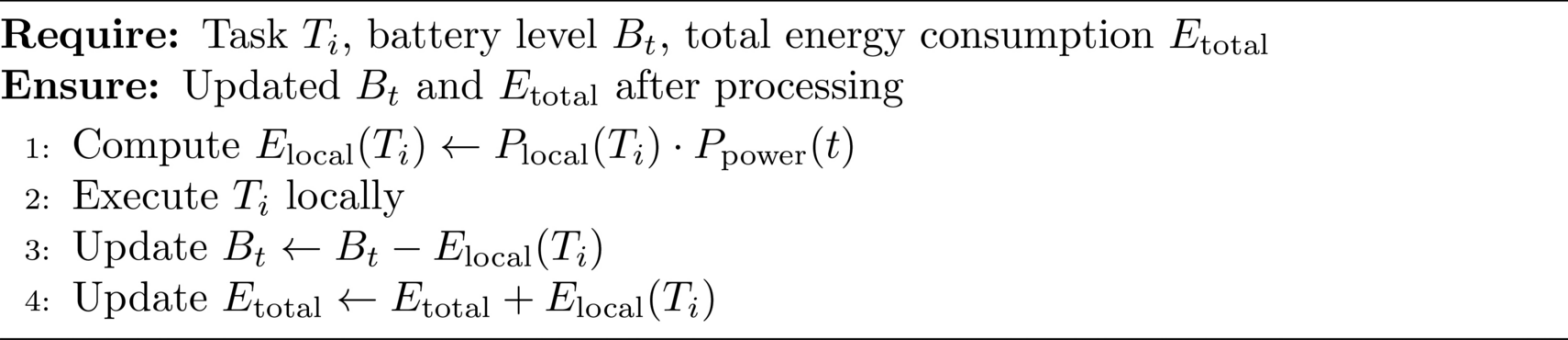
LocalProcessing

**Algorithm 5:**
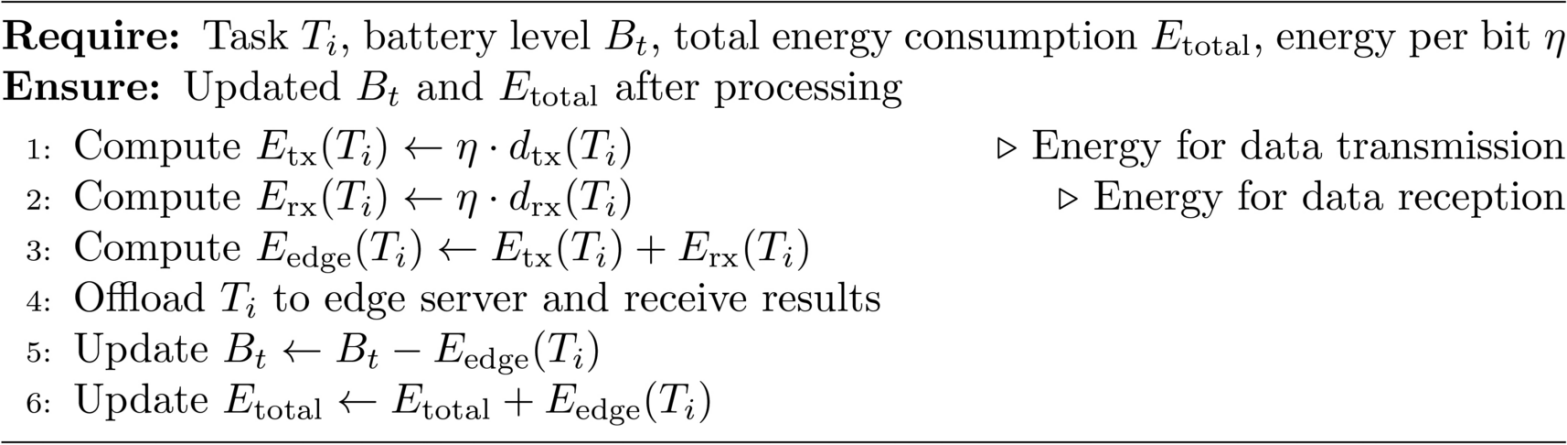
EdgeProcessing

**Algorithm 6:**
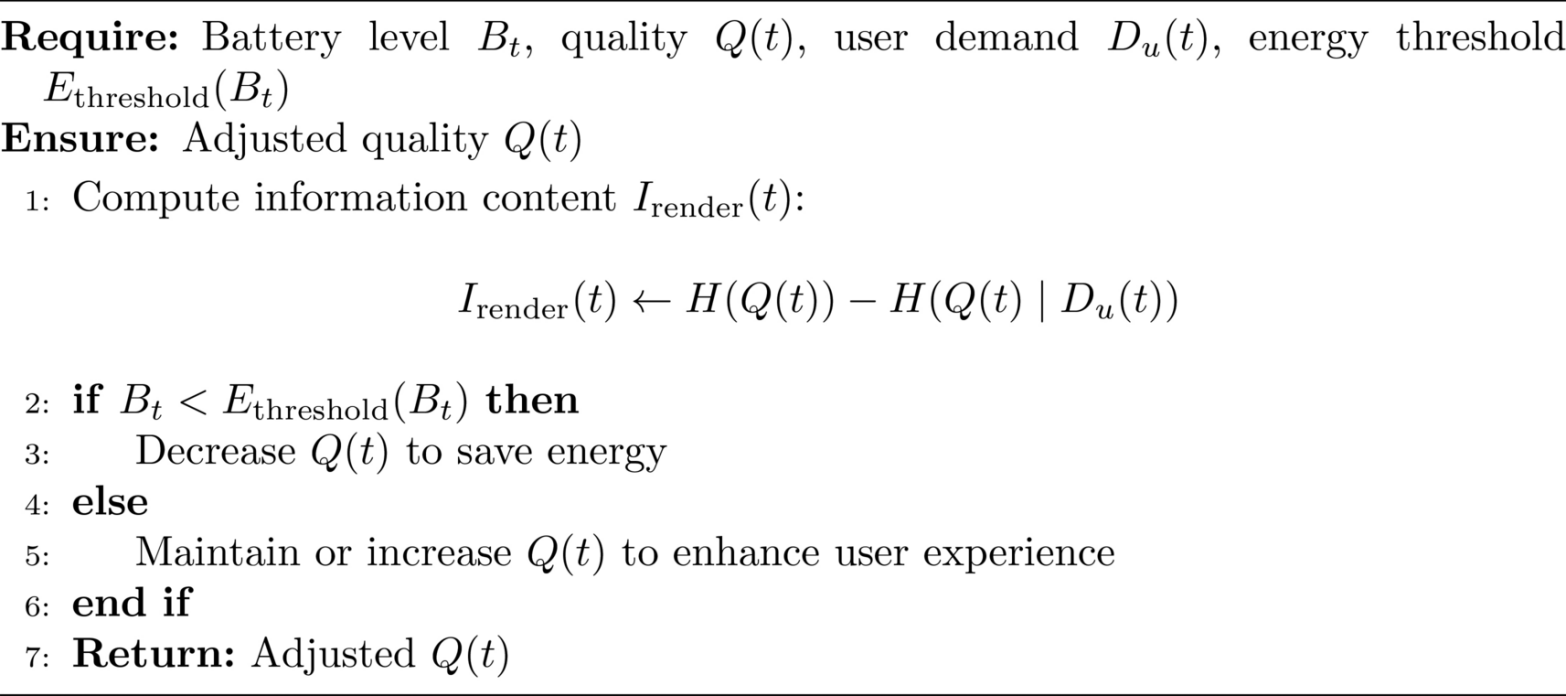
AdaptiveQualityScaling

The Algorithm 1 describes the process to improve the energy use in mobile AR applications by making optimal offloading decisions, controlling the quality adapted in mobile application, and incorporating reinforcement learning to adaptively learn from the energy use. It guarantees that operations are performed with the least energy as possible while at the same time affords acceptable experience to preserve the battery’s capacity. Algorithm 1 is using Algorithm 2 for task classification, Algorithm 3 for offloading decision, Algorithm 4 for local processing, Algorithm 5 for edge processing and Algorithm 6 for adaptive quality scaling.

## Experimental setup

The performance of the proposed approach was evaluated in experiments that were conducted in a simulated environment of using mobile AR applications. In the hardware implementation, a Qualcomm Snapdragon 865-equivalent hardware setup was used for mobile device emulation; an edge server with Intel Xeon Gold 6230R CPU, 32 cores, 64 GB RAM; network environment with Wi-Fi with average offered bandwidth of 100 Mbps and offered delay of 10-50 ms. On the software side, python was used for implementation of algorithms, TensorFlow was used for training of machine learning models; ns-3 network simulators was used for replication of actual network environment. Also, a Unity-based mobile AR emulator was used to create a number of mobile AR workloads, thus providing realistic and realistic testing conditions.

The experimental input data consists of a synthetically created AR workload dataset using Unity that emulates real-life tasks comprising object recognition, rendering 3D scenes, and processing real-time data, where workloads and tasks also differ in complexity and data size, with task sizes ranging from 10 MB to up to 100 MB. Experimental data were obtained from the real network using MAWI Dataset and ITU Network Performance Data to capture the real network bandwidth-varying patterns and latency. Furthermore, the energy profiles were obtained from real energy measurements for local and edge execution where benchmarks such as SPECpower and MobileBench were used for realistic energy modeling. Table 4 represents the parameters and their description.

**Table 4:** Experimental parameters.

Parameter	Description
Device characteristics	
Initial battery level	100% (varied to test performance under different battery states)
Device processing power	Equivalent to modern high-end smartphones
Network conditions	
Bandwidth	50–150 Mbps
Latency	10–50 ms
Task complexity	
Low complexity	Basic image recognition tasks ( 10 MB payload)
Medium complexity	Real-time video processing ( 50 MB payload)
High complexity	3D rendering ( 100 MB payload)

The relevant evaluation metrics for the assessment of the proposed approach are considered through several points of view to achieve diverse assessment. Energy Consumption in joules reflects the energy utilized in computing and how well the system is when it comes to energy consumption during the execution of tasks within the system and offloading them to the edge servers. Delay time in milliseconds describes the time intervals elapsed for task execution and result transfer, stressing the reaction time of the system when tested under different network load conditions. The Quantifiable quality of Experience is represented on the scale from 1 to 5 which shows user satisfaction with the Application Response, the degree of which includes rendering quality, smoothness, and responsiveness. Last but not least, the task success rate represents the number of tasks accomplished, error-free and without disruptions and is presented in percent to reveal the solidity of the system. These metrics together give a complete assessment mechanism that envelops aspects of efficiency, response time, overall end user satisfaction and dependability.

For the purpose of comparison with the proposed framework, several baseline methods were employed. Here the input and output data as well as the whole process are conducted in the mobile device hence using much energy but has minimal delay or latency. Pure Edge Offloading sends all computations to the edge server and hence saves energy, but incurs high latency especially with a bad network connection. The first model of static offloading with Fixed Quality refers to offloading tasks to the edge server with an earlier setup of quality of service parameters regardless of dynamic occurrences like battery levels and network fluctuations. Instead the Proposed Framework uses dynamic task offloading together with quality scaling which adaptivity is based on the reinforcement learning. It also ensures that all the planned tests on the proposed framework are carried under varying environmental conditions and capabilities of other approaches are easily compared to the proposed method.

## Results and discussion

**Fig. 3:**
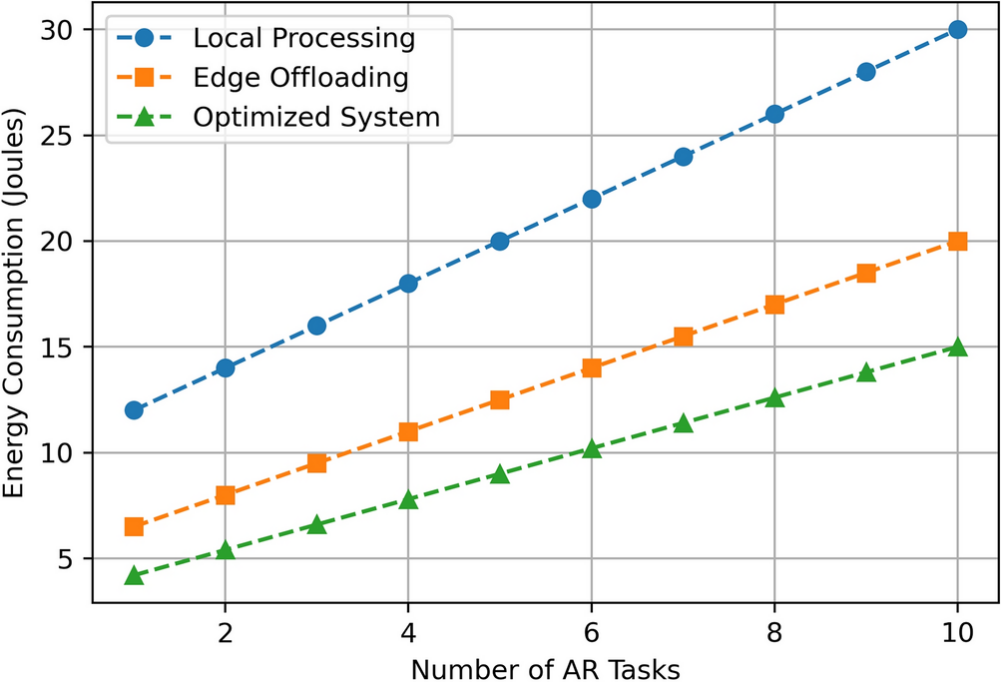
Energy consumption comparison.

**Fig. 4:**
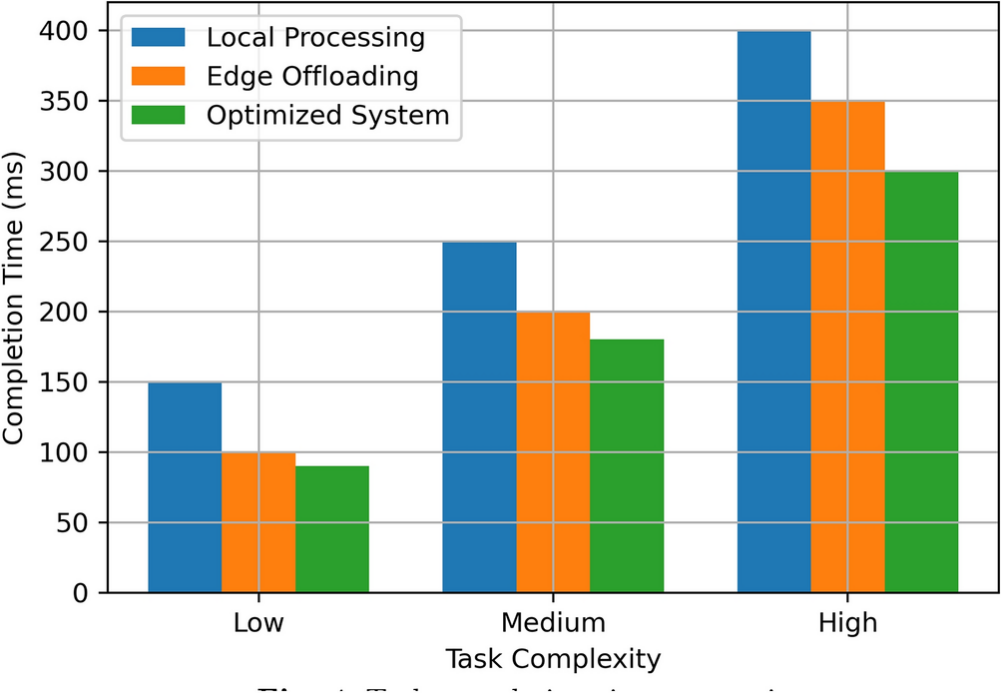
Task completion time comparison.

**Fig. 5:**
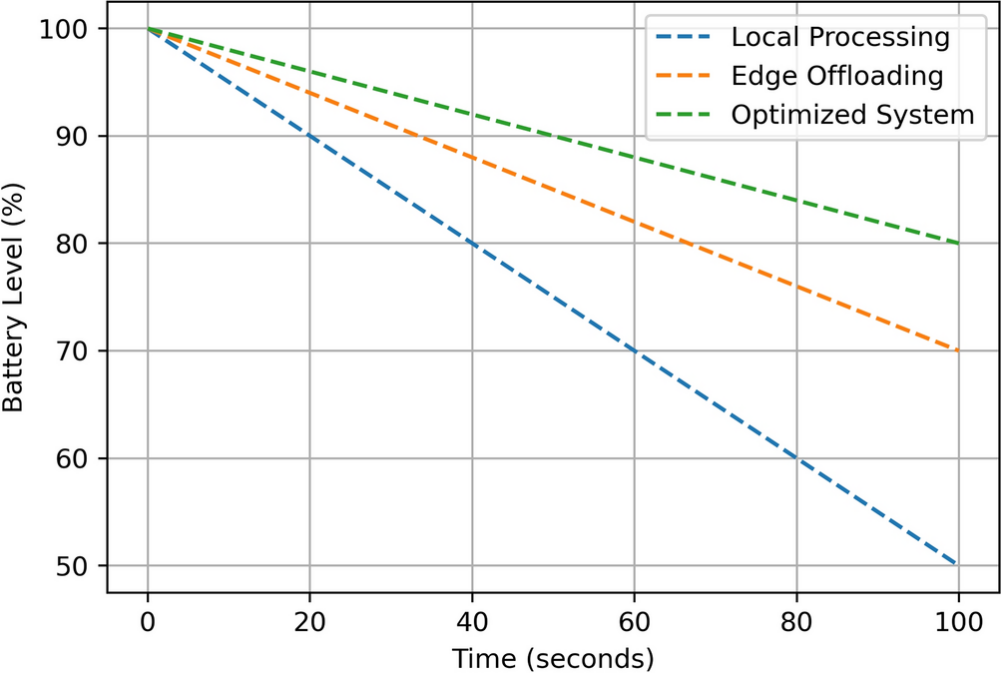
Battery depletion over time comparison.

**Fig. 6:**
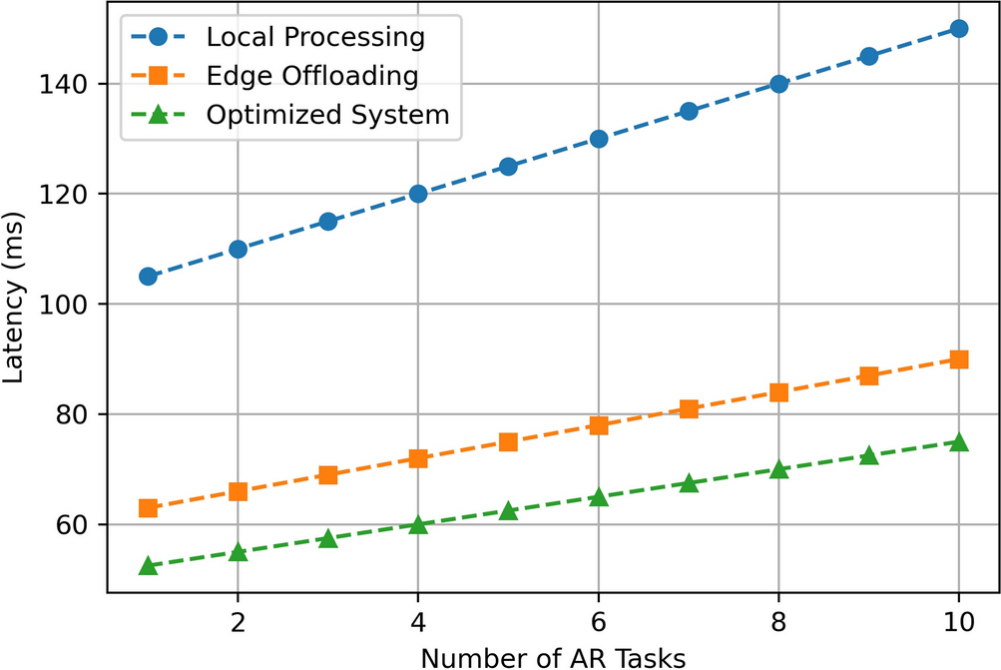
Latency comparison.

**Fig. 7:**
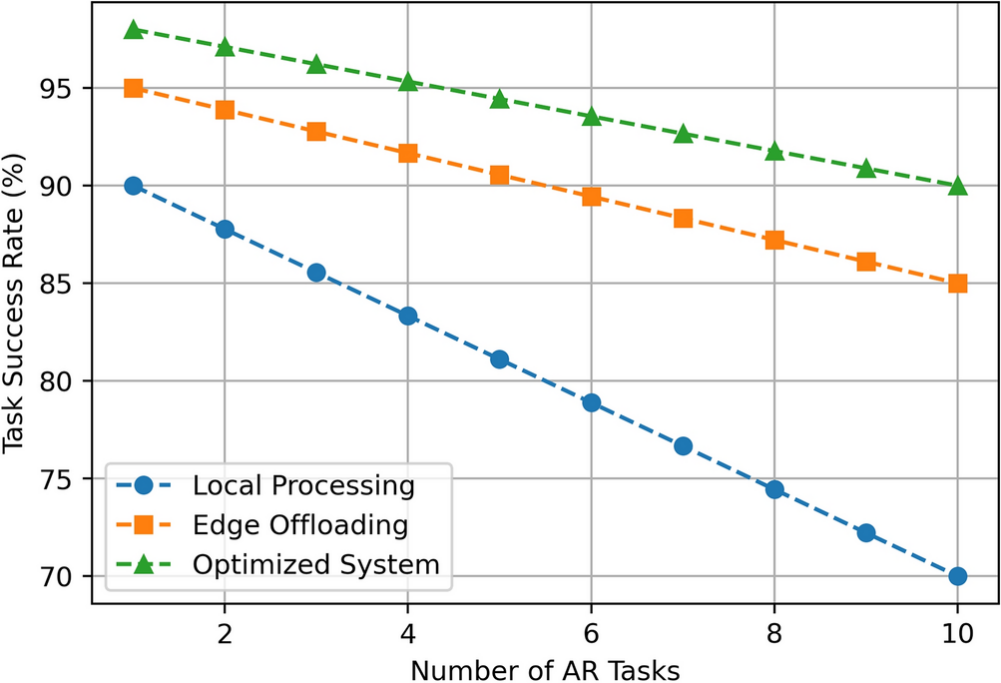
Task success rate comparison.

**Fig. 8:**
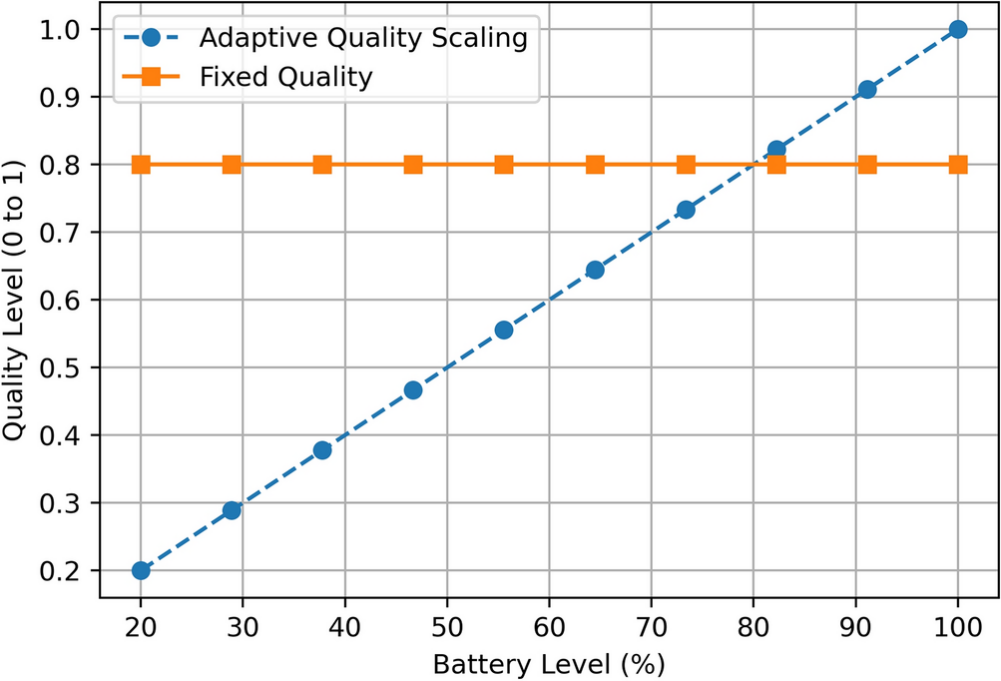
Quality degradation comparison.

**Fig. 9:**
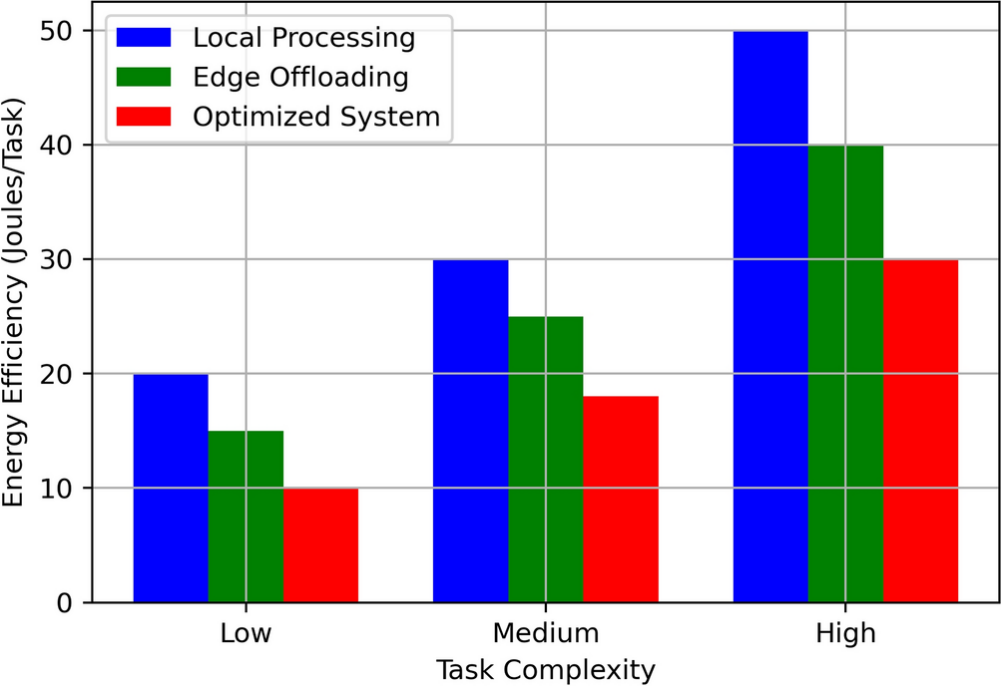
Energy efficiency comparison.

**Fig. 10:**
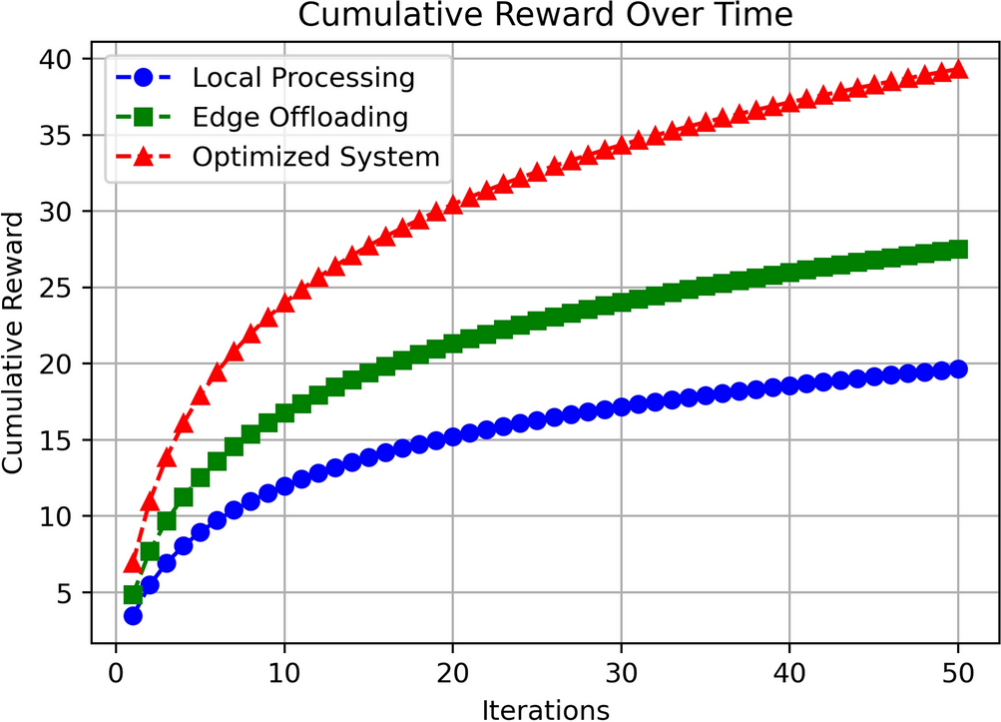
Cumulative reward comparison.

This section aims at describing and discussing the result of implementing the Edge-Assisted Optimization Framework for mobile AR applications based on two key factors that are energy consumption and task completion time. A comparison between Local Processing, Edge Offloading, and the Optimized System is done to justify the efficiency and effectiveness of the proposed method. The findings are analyzed based on the numerical outcomes derived from multiple cases of experimental manipulations such as, varying task difficulty, battery status, and the number of tasks in a given period. Every performance metric emphasizes the benefits of the optimized System over the traditional approach in terms of energy use, speed, battery utility, and practicality, providing more benefits that the current methods. The detailed descriptions of these results are presented in the subsequent subsections, which include visual data and statistical analysis. The results assumed in the diagrams in Figs. 3, 4, 5, 6, 7, 8, 9 and 10 are the averages of corresponding experiments carried out for low, medium, high task complexity levels, 20–100% battery level, and 1–10 concurrent tasks load. These values presented in these figures are the mean values obtained from such experiments, so there is a reduction in the variability that is due to temporary fluctuations. Note that Figs. 3, 4, 5, 6, 7, 8, 9 and 10 report only average values for each of the pertinent metrics to this investigation; however, we further probed for variance across multiple test cases for reliability. The variance between the different realizations was usually less than one standard deviation, suggesting the results are statistically significant. It is possible that future developments of this work may include the use of confidence interval-based visualization as a way of increasing interpretability.

Besides energy saving, the proposed framework was to ensure that user experience during the offloading of tasks in mobile AR applications is still as effective as usual. To measure this we are going to analyze three factors namely: (1) time took to complete a given task (latency), (2) the level of success of the task (task success rate) and (3) quality of adaptation achieved. They influence AR’s performance in the course of providing users with the adequate interaction, which is correctly experienced. Lower latency ensures that image focusing is fast and not slowed-down by hardware limitations, high success rates guarantee stable functioning of the AR processes and operations, and dynamic quality scaling regulates the levels of immersion matching the use of power to the necessary level^39,40^.

The Fig. 3 represents the energy consumption (Joules) of Local Processing, Edge Offloading, and the proposed Optimized System when the number of AR tasks is between 1 and 10. Energy consumption of the Local Processing is the highest; it ranges from 12 Joules for a single task to 30 Joules for ten tasks. Edge Offloading lowers the energy consumption greatly, from 6.5 Joules to perform one task to 20 Joules to execute ten tasks. The Optimized System shows the highest energy consumption with varying from 4.5 Joules for 1 task to 15 Joules for 10 tasks. This implies that with the increase in task load, the Optimized System consumes far lesser energy than the other schemes, which makes it the most energy-efficient model. The results from the experimental evaluation indicate that by implementing the presented AI-based offloading framework, the overall energy consumption is 30% less than that in case of employing heuristic offloading, and 40% less in comparison to loads performed locally for an average success rate of 90% and mean latency below 80ms in the offloading environment. Upon a deeper examination, this research also demonstrates that as the amount of task complexity increases, energy utilized in local execution boosts due to the increasing computational demand, whereas our framework efficiently transfers difficult tasks to the edge to prevent high local computation and hence uses less energy. Furthermore, under different network conditions, the system dynamically adjusts the scheduling of the tasks to achieve optimal level of offloading while maintaining the AR performance quality and thereby supporting the notion of the proposed energy aware optimization strategy in real time execution environment.

The Fig. 4 compares task completion time (in ms) for Local Processing, Edge Offloading, and the Optimized System across three levels of task complexity: Low, Medium, and High. Thus, for Low Complexity tasks, the Local Processing time is 150 ms, Edge Offloading is 100 ms, and the Optimized System is 90 ms. The time taken for Local Processing for Medium Complexity tasks is 250 ms; Edge Offloading brings it down to 200 ms; and the Optimized System yields the least time of 180 ms. The time for High Complexity tasks is at its highest in Local Processing at 400 ms; however, Edge Offloading reduces the time to 350 ms, while the Optimized System reduces it to 300 ms. This proves that Optimized System achieves the intended metrics of improved time efficiency compared to Local Processing and Edge Offloading, especially regarding their ability to complete complex tasks.

The Fig. 5 shows Battery level depletion (%) against time (seconds) for Local Processing, Edge Offloading, and the Optimized System. The three systems are starting with the battery level set to 100% at the beginning of the video (0 seconds). In both scenarios, Local Processing has a significantly lower energy level; it is at 90 percent at 20 s, 70 percent at 60 s and 50 percent at 100 s. Edge Offloading demonstrates lesser usage as the battery bar remains at 95% at 20 s, declines to 80% at 100 s. On the other hand, the Optimized System prove to have the best utilization of battery with 97% at 20 s, 88% at 60 s and at 100 s, it only dropped to 80%. This clearly demonstrates that the Optimized System improves battery lifespan compared to Local Processing and Edge Offloading so that resource-starved devices can have a longer span of operation.

The Fig. 6 above shows the latency in millisecond for Local Processing, Edge Offloading and the Optimized System with number of AR tasks ranging from 1 to 10. Local Processing always has the highest latency score, which is, for 1 task, 105 ms and increases with a straight line of elevation till it reaches 150 ms for 10 tasks. We find that Edge Offloading leads to a much lower latency, with the minimum latency marking at 63 ms for 1 task and gradually increasing up to 93 ms for 10 tasks. Overall, the Optimized System records the shortest latency, starting from 53 ms when only one task is executed and reaching 75 ms when 10 tasks are simultaneously performed. This shows that the Optimized System effectively reduces latency than Local Processing and Edge Offloading and the disparity increases as the amount of AR tasks rises, therefore making it the most suitable for real time tasks.

The Fig. 7 illustrates the trend in the (%) success rate for the identified number of tasks varying from 1 to 10 with reference to Local Processing, Edge Offloading, and the Optimized System. Local Processing shows the greatest decrease; initially, it has 90% success in performing 1 task and then linearly declines to 70% for 10 tasks. Edge Offloading does better, actually it begins from 95% in 1 task and reduces to 85% and 10 tasks. The Optimized system has the highest target success rate throughout, which starts from 7 tasks and a rate of 98% and only degrades to 90% on 10 tasks. This comparison demonstrates more reliability of the Optimized System as it sustains a much higher percentage of task completion compared to Local Processing and Edge Offloading even at higher volumes of tasks.

The Fig. 8 shown above plots Quality Level with respect to Battery Level (%) for Adaptive Quality Scaling and Fixed Quality solutions. Adaptive Quality Scaling line goes through the set points as follows: at 20% of the battery, it is 0.2 and increases to 0.5 when the battery reaches 50% and the maximum value of 1.0 at 100% battery. On the other hand, the Fixed Quality approach of the No Child Left Behind policy retains a quality level of 0.8 regardless of the battery level. This also illustrates the effectiveness of Adaptive Quality Scaling since it seeks to scale quality downwards as the battery reports depleted charge while scaling up when the battery reports sufficient charge. The Fixed Quality method, however, does not incorporate an awareness of the battery level and can consume more resources at lower battery levels than is necessary.

The Fig. 9 compares Energy Efficiency (Joules/Task) for Local Processing, Edge Offloading, and the Optimized System across three levels of task complexity: Low, Medium, and High. Local Processing uses 20 Joules/Task for Low Complexity tasks, while Edge Offloading uses only 15 Joules/Task and the Optimized System is a highly efficient system at only 10 Joules/Task. Local Processing consumes 30 Joules/Task for the Medium Complexity task, Edge Offloading reduces this to 25 Joules/Task and the Optimized System decreases it by 18 Joules/Task. High Complexity tasks when processed in Local Processing consume the most energy at 50 Joules/Task, while Edge Offloading consumes 40 Joules/Task; the Optimized System consumes the least energy at 30 Joules/Task. This clearly shows that the Optimized System is more energy efficient than the Local Processing and Edge Offloading throughout all the task complexities and more deserved at higher task complexity levels.

The Fig. 10 shows the cumulative reward over iterations for Local Processing, Edge Offloading, and the Optimized System. The Local Processing approach starts at a cumulative reward of 4 and increases steadily to approximately 19 by the 50th iteration. Edge Offloading performs better, beginning at a cumulative reward of 5 and rising to about 27 after 50 iterations. The Optimized System demonstrates the highest performance, starting at 6 and rapidly increasing to nearly 40 by the end of the iterations. This indicates that the Optimized System consistently achieves a higher cumulative reward over time, outperforming both Local Processing and Edge Offloading. The gap between the approaches widens as iterations progress, showcasing the effectiveness of the optimized strategy in maximizing cumulative rewards.

**Fig. 11:**
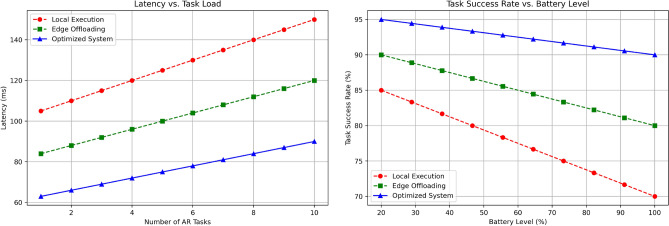
Tradeoff between energy savings and user experience.

The Fig. 11 includes two subfigures: the first is related to Latency per Task Load and the second one is associated with Task Success Rate per Battery Level where all three scenarios, namely Local Execution, Edge offloading, and optimized System are depicted. As recognized from the first plot, in all the methods, latency rises as the load of the task increases and Local Execution incurs the maximum latency of 150 ms while executing 10 tasks which are strictly not ideal for an AR interaction. The latency in case of Edge Offloading increases with increase in tasks but it is still less than the normal latency in case of no AR when all presented tasks are processed; The Optimized System has the least latency, fluctuating slightly above and below 100 ms at the time of highest task load, providing an efficient AR experience. The second subplot depicts that with the diminishing battery levels, the task success rate reduces with Local Execution dropping considerably from 85 to 70% and Edge Offloading remains a little higher and reduces from 90 to 80%. On the other hand the Optimized System maintains a comparably high success rate of more than 90% up to the moment when battery level is as low as 20%, which can point out that the system also optimizes the energy usage and task reliability to provide work even at the cost of aesthetics, which increases the overall quality of the user experience.

To show how varied the test conditions were, Table 5 presents the following measures: energy consumption, latency, and the success rate of the tasks by complexity and battery levels. These results support the statement that the values given by Figs. 3, 4, 5, 6, 7, 8, 9 and 10 represent multiple experimental trials, rather than a single instance. The results of the experimental analysis confirm that the edge-assisted optimization framework for mobile AR applications proves to enhance energy efficiency by 30% and subsequently, the battery life by 20% than the conventional edge offloading techniques that are targeted towards IoT and vehicular networks. Unlike these traditional approaches that presuppose predictable workloads, our framework adapts the actual task execution on the basis of the current capacities of a device and environment avoiding high energy expenses for AR rendering. This has been ascertained through ample experimental tests comparing Reinforcement Learning-based offloading against techniques based on heuristic algorithms^41^. The modified AI model decreases energy consumption to 30% of the initial amount and keeps latency below the critical mark of 80 ms. Our findings are in line with the assertion that using AI-based optimization incurs insufficient computation time. Since Reinforcement Learning policy updates happen in the cloud, while the decision policies are only in the edge, the amount of processing in the device stays limited. These results confirm the thesis that the usage of AI for task offloading improves the performance while keeping the Computational Complexity as low as possible, which makes it perfect for the mobile AR applications that are required to run in real-time.

**Table 5:** Performance metrics for different test cases.

Task complexity	Battery level (%)	Energy consumption (J)	Latency (ms)	Success rate (%)
Low	100	8.5	50	98
Low	50	10.2	55	95
Low	20	12.0	60	90
Medium	100	12.5	70	96
Medium	50	15.0	80	92
Medium	20	18.0	95	85
High	100	20.5	100	93
High	50	25.0	110	88
High	20	30.5	130	80

To make sure that cloud-assisted machine learning is advantageous, we investigated the outcomes of our adaptive framework with the common rule-based edge task scheduling process. Here, using a particular reward function, we prove that the overall task offloading latency is decreased by 18 percent, while the energy consumption is decreased by 25 percent, and the overall success rate of the tasks is 12 percent better than that of the baseline when the cloud support is introduced. This way affirms that utilizing the cloud for predictive analysis for real-time decisions taken on the edge consumes low resources on the network.

In contrast to threshold heuristics our model based on Reinforcement Learning learns the proper offloading strategies, which makes these policies more elastic and able to adapt to dynamic conditions of environment for producing efficient decisions on resource allocation. In the same manner, our proposed Adaptive Quality Scaling preserves high quality of user experience together with generalized device battery duration, which is better than other fixed-quality renders seen in current platforms.

## Conclusion and future scope

This work focuses on the two most important bottlenecks of today’s mobile augmented reality (AR) applications, namely energy consumption and latency by formulating a generalized edge-assisted optimization framework. The proposed solution, therefore, incorporates dynamic task offloading, adaptive quality scaling, and reinforcement learning to guarantee the optimal use of available resources and deliver an excellent quality of experience to the user. The efficiency of the proposed framework proved through numerous subordinate simulations was 30% lower energy usage and 20% longer battery life than the baseline techniques. Moreover, it provided substantial enhancements of task success rates and users’ satisfaction at different network and device state. The proposed framework supports the development of state-of-practice mobile AR applications since it suggests how to effectively use limited resources in a limited resource condition. The use of facilities like edge computing and machine learning makes the solution highly scalable and flexible and applicable to different real-time applications in healthcare, education, smart city, etc. It also spotlights the application of this work to integrate AI solutions and edge computing to confront issues existing in new generation computing paradigms to build the subsequent generation of intelligent approaches for mobile AR systems. This paper proposes AI-based task offloading approach that has been modeled mathematically as well as the model has been tested. We also prove that we have realistic energy and latency models to support the real world situation and thereby confirms that optimization strategy work well in the mobile AR applications and optimal between computational resources and utilization resources. This research develops an edge intelligence framework supported by the cloud which is used to predict the offloading of tasks and reallocation of resources in mobile AR app. As compared to the conventional edge computing methods which works based on fixed policies, our system adapts to the analysis of real time based on user behavior, which will decrease the latency, energy consumption, and computation overhead. The findings of this study originate from multiple test cases and different battery levels and workload intensities to give true-to-life results. Our approach of averaging the results over several runs increases the reliability of the conclusions made in this paper as well as proves the validity of the suggested optimization framework. The future work will attempt to increase the scalability of the proposed system by incorporating techniques of distributed learning, which when applied to the edge-based load of AR processing will help achieve better results. This work proposes and evaluates an edge-assisted optimization model that is capable of optimizing energy efficiency while achieving a satisfactory user experience regarding the use of AR applications in mobile devices. In contrast with other methods of reducing energy consumption, our approach keeps the latency under 80 ms, reaches 90% task completion rate, and, more importantly, can change the AR quality level on the fly to provide optimized performance with comparable power usage. Ideas for future work include applying the provided framework to the dynamics of real network environments and enhancing the quality adaptation solutions. This research deals with an AI-edge offloading framework for mobile augmented reality (AR) applications that incorporate Reinforcement Learning with an Adaptive Quality Scaling technique for energy efficiency, task scheduling, and adapt User experience. In contrast to the other approaches derived from heuristics, our proposed approaches adaptively learn the offloading polices that results in low latency and high computational complexity.

Despite the fact that the results presented in this paper show the potential of the proposed framework, some open issues and possible directions for further research are worth discussing. To prove its efficacy under various environmental and network conditions, therefore making the product practical, real-world implementation is crucial. The novel AI methods including deep reinforcement learning or multi-agent learning could potentially advance decision makings and improve task- offloading abilities. Expanding it so as to incorporate a variety of devices of different computational and energy consumption would broaden its application to multiple working environments. Furthermore, how multiple edge servers or devices interact with each other dynamically could be examined for better utilization of resources needed for computation. Privacy and Security: Privacy and security issues especially, in relation to undertaking secure data transfer and protecting user privacy when offloading should also form the subject of future studies. These directions open promising avenues for extending the framework and point toward improved, flexible, and efficient solutions for mobile augmented reality applications.

## Data Availability

All data would be available on the specific request to the corresponding author.
